# LSD-stimulated behaviors in mice require β-arrestin 2 but not β-arrestin 1

**DOI:** 10.1038/s41598-021-96736-3

**Published:** 2021-09-03

**Authors:** Ramona M. Rodriguiz, Vineet Nadkarni, Christopher R. Means, Vladimir M. Pogorelov, Yi-Ting Chiu, Bryan L. Roth, William C. Wetsel

**Affiliations:** 1 Department of Psychiatry and Behavioral Sciences, Duke University Medical Center, 354 Sands Building, 303 Research Drive, PO Box 103203, Durham, NC 27710 USA; 2 Mouse Behavioral and Neuroendocrine Analysis Core Facility, Duke University Medical Center, Durham, NC 27710 USA; 3 Division of Chemical Biology and Medicinal Chemistry, Department of Pharmacology, Center for Integrative Chemical Biology and Drug Discovery, Eshelman School of Pharmacy, University of North Carolina at Chapel Hill School of Medicine, Chapel Hill, NC 27599 USA; 4 Division of Chemical Biology and Medicinal Chemistry, Department of Pharmacology and National Institute of Mental Health Psychoactive Drug Screening Program, Center for Integrative Chemical Biology and Drug Discovery, Eshelman School of Pharmacy, University of North Carolina at Chapel Hill School of Medicine, Chapel Hill, NC 27599 USA; 5 Departments of Cell Biology and Neurobiology, Duke University Medical Center, Durham, NC 27710 USA

**Keywords:** Drug discovery, Neuroscience

## Abstract

Recent evidence suggests that psychedelic drugs can exert beneficial effects on anxiety, depression, and ethanol and nicotine abuse in humans. However, their hallucinogenic side-effects often preclude their clinical use. Lysergic acid diethylamide (LSD) is a prototypical hallucinogen and its psychedelic actions are exerted through the 5-HT_2A_ serotonin receptor (5-HT2AR). 5-HT2AR activation stimulates Gq- and β-arrestin- (βArr) mediated signaling. To separate these signaling modalities, we have used βArr1 and βArr2 mice. We find that LSD stimulates motor activities to similar extents in WT and βArr1-KO mice, without effects in βArr2-KOs. LSD robustly stimulates many surrogates of psychedelic drug actions including head twitches, grooming, retrograde walking, and nose-poking in WT and βArr1-KO animals. By contrast, in βArr2-KO mice head twitch responses are low with LSD and this psychedelic is without effects on other surrogates. The 5-HT2AR antagonist MDL100907 (MDL) blocks the LSD effects. LSD also disrupts prepulse inhibition (PPI) in WT and βArr1-KOs, but not in βArr2-KOs. MDL restores LSD-mediated disruption of PPI in WT mice; haloperidol is required for normalization of PPI in βArr1-KOs. Collectively, these results reveal that LSD’s psychedelic drug-like actions appear to require βArr2.

## Introduction

Lysergic acid diethylamide (LSD) is a prototypical psychedelic drug and is one of the most potent drugs in this class^1^. LSD alters sensation, perception, thought, mood, sense of time and space, and consciousness of self in humans^1,2^. Since LSD-induced states bear many similarities to early acute phases of psychosis^2^ and because serotonin (5-HT) and LSD both contain an indolamine moiety, Woolley and Shaw^3^ proposed that aberrant 5-HT levels in brain may produce mental disturbances including psychosis. This suggestion gave rise to the 5-HT hypothesis for schizophrenia and stimulated researchers to study LSD in hopes of gaining a better understanding of the disorder. However, this research was largely curtailed when LSD was classified as a DEA Schedule I drug in the 1960’s. Recent research has revealed that LSD has medicinal value in treating cluster headaches^4^, anxiety and depressive disorders in life-threatening conditions when combined with psychotherapy^5^, and it may have potential for studying human consciousness and substance abuse^6,7^.

LSD shares structural similarities to 5-HT^1^. Thus, it is not surprising that LSD has high affinities for all thirteen 5-HT G protein coupled receptors (GPCRs)^8–10^. Besides 5-HT receptors, LSD activates other biogenic amine GPCRs^8^ and this polypharmacology may contribute to LSD’s many actions. One activity in particular regarding LSD is its hallucinogenic actions. This activity is ascribed to 5-HT_2A_ receptor (5-HT2AR) stimulation since in drug discrimination studies, discrimination-derived potency is correlated with hallucinogenic potency in humans^11^. Because the same psychedelics produce head twitches in mice, this response is used as a proxy for hallucinations in humans^12^, even though non-psychedelic drugs like 5-hydroxytryptophan induce robust head-twitch responses (HTRs)^13^. Hallucinogen-induced HTRs in rodents are blocked by the highly selective 5-HT2AR antagonist MDL100907^14–16^ and are absent in *htr2A* knockout (KO) mice^17,18^. In addition, human studies have shown the hallucinogenic actions of LSD are blocked with the 5-HT2AR preferring antagonist ketanserin^19^. Thus, the hallucinogenic effects of LSD appear to be mediated through the 5-HT2AR^20^.

The 5-HT2AR is a rhodopsin family member of GPCRs that is coupled to G_q_ protein and to non-visual arrestin mediated signaling^21–24^. Recent experiments reveal the 5-HT2AR preferentially activates G_q_ family members, with moderate activity at G_z_, and minimal activities at G_i_-, G_12/13_-, and G_s_-family members^25^. However, the 5-HT2AR binds to both β-arrestin 1 (βArr1) and βArr2 proteins in vitro and is complexed with these βArrs in cortical neurons *in vivo*^24^. Note, within the arrestin family the non-visual βArr1 and βArr2 are termed Arr2 and Arr3, respectively. While most GPCR agonists, like 5-HT, activate both G protein and βArr signaling, ligand binding can activate also G protein-dependent signaling while serving to activate or inhibit βArr-mediated signaling. Hence, a given ligand can act as an agonist at one pathway while inhibiting the other pathway or it can possess combinations of these actions. This property is termed functional selectivity or biased signaling^26–28^ and ligands have been developed to exploit these signaling features^29^. Although LSD activates G protein signaling at many GPCRs^10^, this psychedelic stimulates βArr-mediated responses at most tested biogenic amine GPCRs^8^. Interestingly, LSD displays βArr-biased signaling at the 5-HT2AR^9,10,25^. Most 5-HT2AR-containing neurons express both βArr1 and βArr2^24^, and global βArr1 and βArr2 knockout (KO) mice have been generated^30,31^. Since LSD is βArr biased at the 5-HT2AR, the present investigations were conducted to determine whether LSD produces behavioral effects that were differential among the wild-type (WT) and βArr1-KO, and WT and βArr2-KO mice.

## Results

The *Arrb1* (βArr1 protein) or *Arrb2* (βArr2 protein) genes were obtained from 129 libraries, the constructs were injected into ES cells that were microinjected into C57BL/6 blastocysts^30,31^. These chimeric mice were backcrossed to C57BL/6J mice. Both the βArr1 and βArr2 mice are on a C57BL/6J genetic background and are maintained as separate strains. However, their behavioral responses are somewhat different between the strains. All experiments have an approximate equal mix of mutant and relevant WT littermates. No sex effects were detected in any experiments.

### Effects of *Arrb1* or *Arrb2* deletion on LSD-stimulated motor activities

LSD has been reported to stimulate, inhibit, or produce biphasic effects on a variety of motor activities in rodents^17,32–36^. We examined responses to LSD in the global βArr1-KO and global βArr2-KO mice to determine whether disruption of either gene product could modify the behavioral responses to this hallucinogen and to test whether 5-HT2AR antagonism could block these effects. Locomotor, rearing, and stereotypical activities were monitored at 5-min intervals over the 120 min test in both the βArr1 and βArr2 genotypes (Supplementary Figures S1−S2).

**Figure 1:**
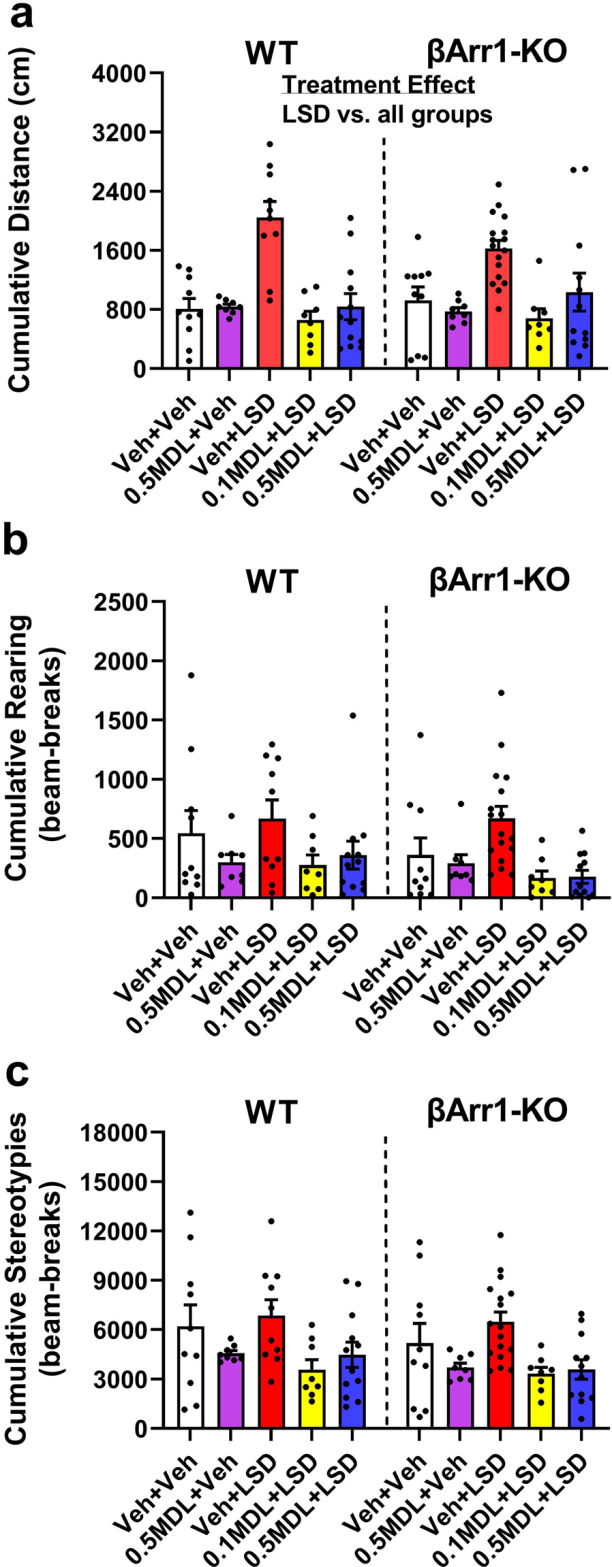
Effects of LSD and MDL100907 on cumulative motor activities in β-arrestin 1 mice. Mice were administered the vehicle or different doses of MDL100907 (MDL) and placed into the open field for 30 min. They were removed, injected with the vehicle or 0.3 mg/kg LSD, and immediately returned to the test arena for 90 min. The cumulative baseline motor activities (0–30 min) are presented in Supplementary Table S1. A two-way ANOVA failed to identify any significant effects for baseline locomotion; separate two-way ANOVAs detected significant treatment effects for baseline rearing [F(4,93) = 6.943, *p* < 0.001] and stereotypical activities [F(4,93) = 7.110, *p* < 0.001]. To control for these baseline differences in rearing and stereotypy, the LSD post-injection activities were analyzed by ANCOVA. (**a**) LSD-stimulated locomotor activities in WT and βArr1-KO mice. A two-way ANOVA identified a significant treatment effect [F(4,93) = 16.916, *p* < 0.001]. (**b**) LSD-stimulated rearing activities in βArr1 animals. An ANCOVA failed to find any significant differences. (**c**) LSD-stimulated stereotypical activities in βArr1 mice. An ANCOVA revealed a significant treatment effect [F(4,92) = 7.029, *P* = 0.024]. N = 8–17 mice/group. Bonferroni corrected *post-hoc* tests for locomotion for treatment effects: *p* < 0.001, LSD vs. all groups.

When cumulative baseline locomotion was examined in βArr1 mice, activity was not differentiated by genotype or by the pre-assigned treatment condition (Supplementary Table S1). Following LSD injection, only treatment effects were found (Fig. 1a). Here, locomotor activities were stimulated by LSD relative to control groups given the vehicle or 0.5 mg/kg MDL alone (*p* values ≤ 0.001). When administered with LSD, both doses of MDL blocked the locomotor-stimulating effects of this psychedelic.

An examination of cumulative baseline rearing and stereotypical activities in the βArr1 mice found these overall responses to be significantly lower or higher in the vehicle, LSD, 0.1 mg/kg MDL plus LSD, and 0.5 mg/kg MDL plus LSD pre-assigned treatment groups than in the pre-assigned 0.5 mg/kg MDL group (*p* values ≤ 0.001) (Supplementary Table S1. To correct for these baseline differences in the subsequent LSD-post injection analyses for βArr1 mice, their rearing and stereotypical data were analyzed separately by ANCOVA. No significant effects of LSD were observed for rearing (Fig. 1b). By comparison for stereotypical activities, ANCOVA revealed a significant main effect of treatment in βArr1 mice following LSD administration (*p* = 0.024). Nevertheless, Bonferroni *post-hoc* analyses only identified a trend between the group treated with LSD and the group given MDL alone (*p* = 0.062) (Fig. 1c). Collectively, these results indicate that LSD stimulates locomotor activities to similar extents in the WT and βArr1-KO animals, and the 5-HT2AR antagonist MDL blocks these responses. Rearing and stereotypical activities are unaffected by LSD in either genotype.

**Figure 2:**
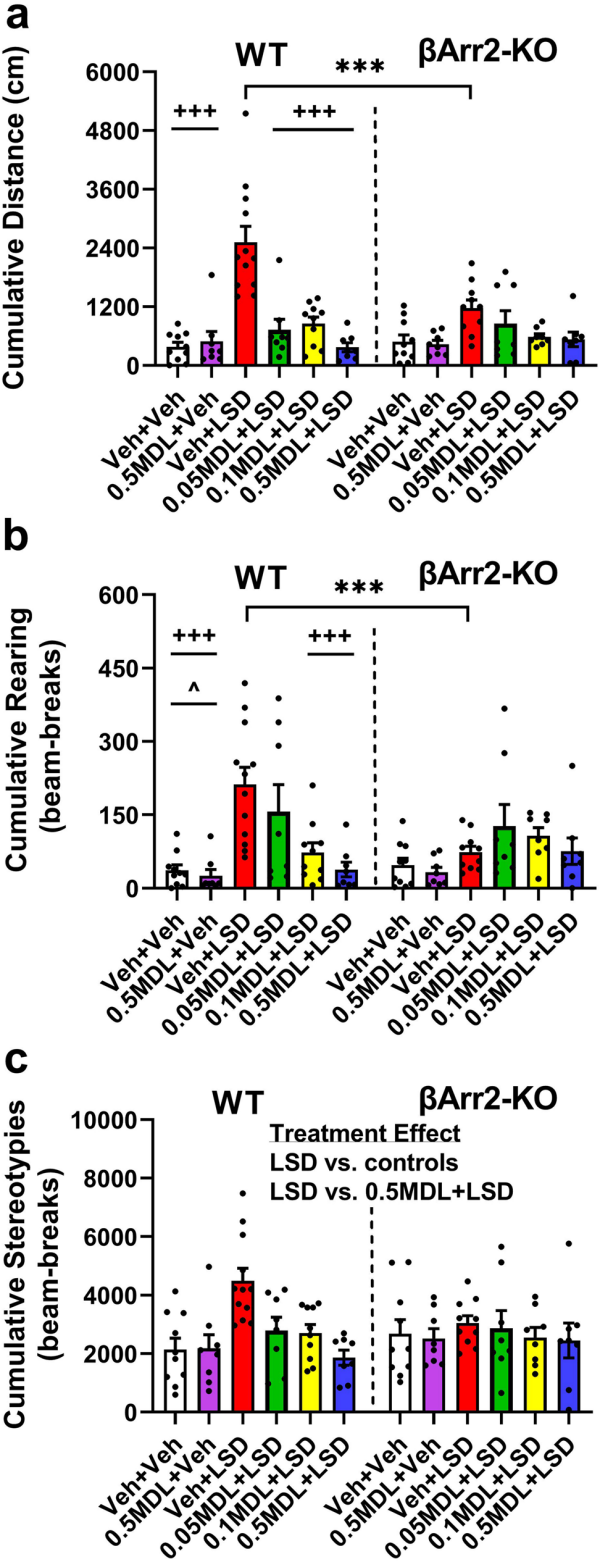
Effects of LSD and MDL100907 on cumulative motor activities in β-arrestin 2 mice. A description of the experimental design is provided in the legend for Fig. 1. The cumulative baseline results (0–30 min) are shown in Supplementary Table S2. Two-way ANOVAs failed to identify any significant effects for baseline locomotion, rearing, or stereotypy. (**a**) LSD-stimulated locomotor activities in WT and βArr2-KO subjects. A two-way ANOVA reported a significant treatment effect [F(5,96) = 18.578, *p* < 0.001] and a significant genotype by treatment interaction [F(5,96) = 5.273, *p* < 0.001]. (**b**) LSD-stimulated rearing activities in βArr2 animals. A two-way ANOVA observed a significant treatment effect [F(5,96) = 7.150, *p* < 0.001] and a significant genotype by treatment interaction [F(5,96) = 3.437, *p* = 0.007]. (**c**) LSD-stimulated stereotypical activities in βArr2 mice. A two-way ANOVA identified a significant treatment effect [F(5,96) = 4.242, *p* = 0.002]. N = 8–12 mice/group. ***p < 0.001, WT vs. KO; ^+++^*p* < 0.001, LSD vs. designated groups within genotype; ^^^*p* < 0.05, 0.05 MDL + LSD vs. controls within genotype. Bonferroni corrected *post-hoc* tests for stereotypy for treatment effects: *p* < 0.05, LSD vs. vehicle and MDL controls; *p* < 0.01, LSD vs. 0.5 MDL + LSD.

When baseline motor activities were evaluated in the βArr2 mice, no significant differences were found (Supplementary Table S2). Effects of LSD in the βArr2-KO mice were quite different from those of the WT animals. LSD was more potent in stimulating cumulative locomotor activities in the WT than in the βArr2-KO mice (*p* values < 0.001) (Fig. 2a). When locomotion was analyzed within WT animals, the LSD-stimulated responses were higher than those in the vehicle and MDL controls, as well as in the treatment groups administered MDL with LSD (*p* values < 0.001). Hence, all three doses of the 5-HT2AR antagonist were efficacious in suppressing the LSD-induced hyperlocomotion to control levels. Although LSD increased locomotor activity in βArr2-KO mice, it was not significantly different from any other treatment group.

Similar to locomotion, LSD also stimulated rearing activities to a greater extent in WT compared to βArr2-KO mice (*p* values < 0.001) (Fig. 2b). In WT animals, vertical activities were increased with LSD over that of the vehicle and MDL controls (*p* values < 0.001). Rearing was higher also in mice given 0.05 mg/kg MDL plus LSD than the controls (*p* values ≤ 0.029). When 0.1 or 0.5 mg/kg MDL was given with LSD, both doses reduced the LSD-stimulated rearing activities to control levels (*p* values ≤ 0.001). By comparison, LSD was without effect in the βArr2-KO mice.

An assessment of stereotypical activities failed to find any genotype differences between the βArr2 mice (Fig. 2c). Nonetheless, treatment effects were evident with LSD stimulating stereotypical activities over that of the vehicle and MDL controls (*p* values ≤ 0.013). Notably, 0.5 mg/kg MDL abrogated the LSD effects (*p* = 0.003) by bringing levels to those of the controls. Together, these results indicate that LSD stimulates locomotor responses in the WT and βArr1-KO animals. LSD stimulated also locomotor, rearing, and stereotypical activities in WT mice from the βArr2 strain. The 5-HT2AR antagonist blocks these LSD-stimulated activities. By striking comparison, LSD failed to significantly stimulate these same motor responses in the βArr2-KO mice above that of the controls.

### LSD effects on additional behaviors

**Figure 3:**
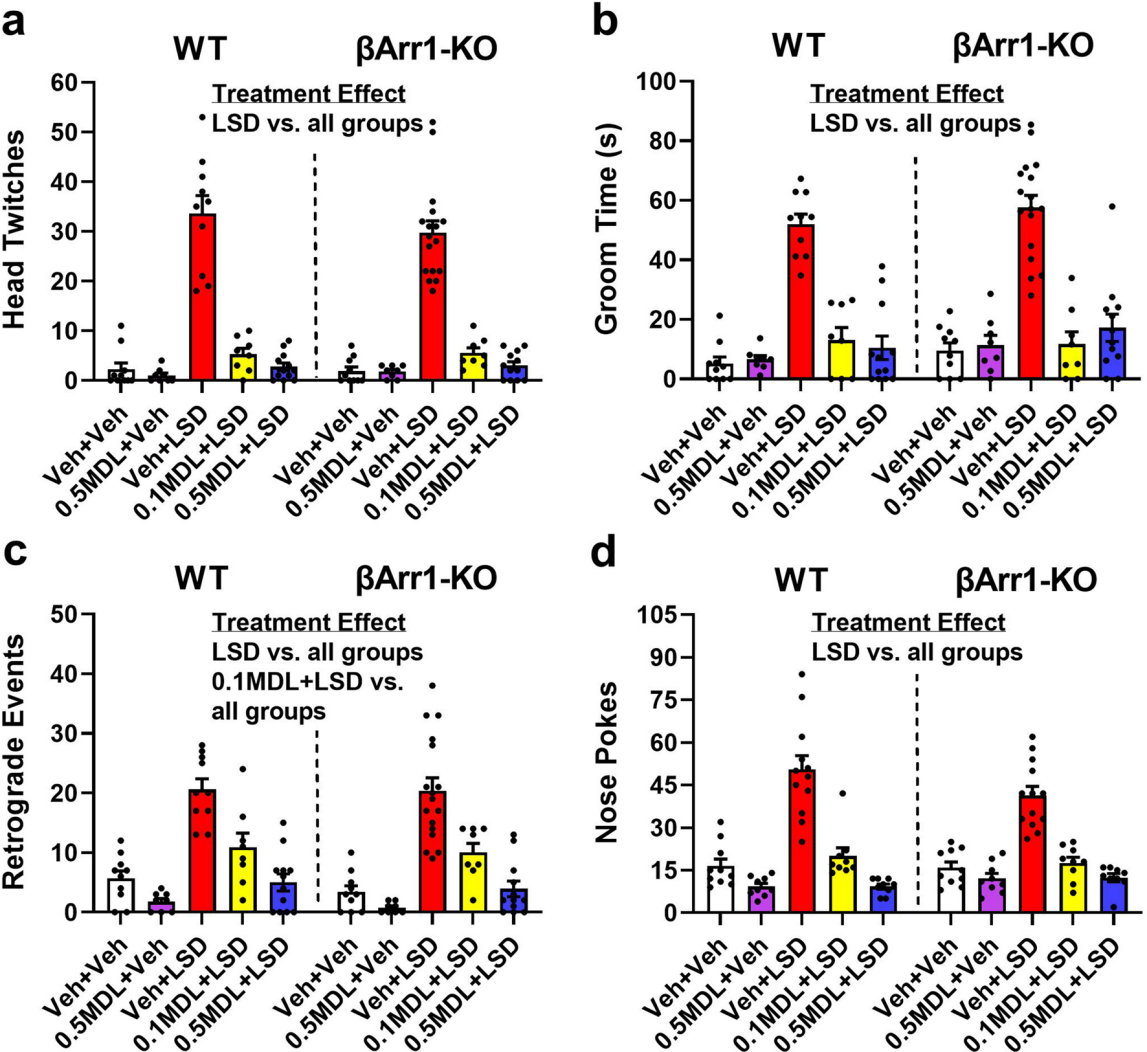
Effects of LSD and MDL100907 on behavioral responses in β-arrestin 1 mice. A description of the experimental design is shown in the Fig. 1 legend. The head twitch, grooming, and retrograde walking results represent the first 30 min after injection of LSD in the open field. Nose poking was examined in a 5-choice serial reaction time apparatus (no rewards) with a similar time-course for the vehicle and MDL injections as in the open field, followed by administration of the vehicle and LSD. (**a**) LSD-stimulated head twitches in WT and βArr1-KO mice. A two-way ANOVA revealed a significant treatment effect [F(4,93) = 114.447, *p* < 0.001]. (**b**) LSD-stimulated grooming in βArr1 animals. A two-way ANOVA identified a significant treatment effect [F(4,93) = 61.232, *p* < 0.001]. (**c**) LSD-stimulated retrograde walking in βArr1 subjects. A two-way ANOVA found the main effect of treatment to be significant [F(4,93) = 43.899, *p* < 0.001]. (**d**) LSD-stimulated nose poking in WT and βArr1-KO mice. A two-way ANOVA observed a significant treatment effect [F(4,89) = 60.656, *p* < 0.001]. N = 8–17 mice/group for head twitch, grooming, and retrograde walking; N = 9–13 mice/group for nose-poking. Bonferroni corrected *post-hoc* tests for head twitch, grooming, retrograde walking, and nose-poking responses for treatment effects: *p* < 0.001, LSD vs. all groups; for retrograde walking for treatment effects: *p* < 0.05, 0.1MDL + LSD vs. all groups.

LSD modifies a number of behaviors in mice^12,17,37–41^ that include, at least, HTRs, grooming, and retrograde walking. When these responses were examined in the βArr1 mice, no genotype differences were noted, although overall treatment effects were evident. Relative to the vehicle and MDL controls, LSD stimulated HTRs, grooming, and nose-poking behaviors in the WT and βArr1-KO mice (*p *values < 0.001) (Fig. 3a,b,d). When 0.1 or 0.5 mg/kg MDL was administered with LSD, both doses of the 5-HT2AR antagonist blocked the LSD effects by restoring the numbers of HTRs, the duration of grooming, and nose-poking behaviors to those of the controls.

Besides HTRs and grooming, LSD was efficacious in potentiating retrograde walking in the WT and βArr1-KO mice compared to the vehicle and MDL controls (*p *values < 0.001) (Fig. 3c). Responses were higher also with 0.1 mg/kg MDL plus LSD than the controls (*p* ≤ 0.018). Nonetheless, both 0.1 and 0.5 mg/kg MDL decreased retrograde walking to control levels when administered with LSD (*p *values < 0.001).

**Figure 4:**
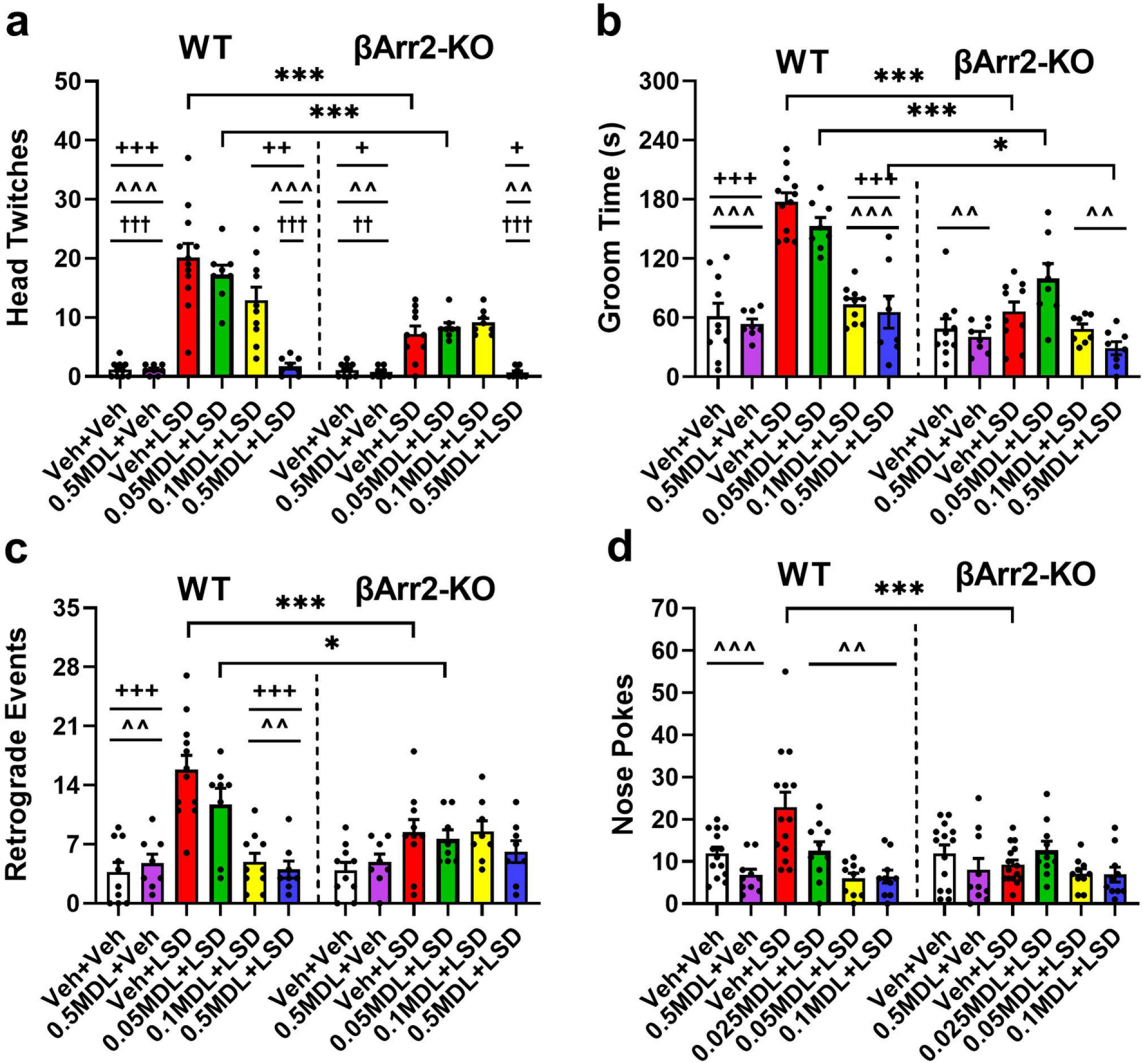
Effects of LSD and MDL100907 on behavioral responses in β-arrestin 2 mice. A description of the experimental design is presented in the Fig. 3 legend. (**a**) LSD-stimulated head twitch responses in WT and βArr2-KO mice. A two-way ANOVA reported significant genotype [F(1,96) = 31.271, *p* < 0.001] and treatment effects [F(5,96) = 41.567, *p* < 0.001]; the genotype by treatment interaction was also significant [F(5,96) = 7.734, *p* < 0.001]. (**b**) LSD-stimulated grooming in βArr2 animals. A two-way ANOVA demonstrated significant genotype [F(1,96) = 51.972, *p* < 0.001] and treatment effects [F(5,96) = 27.987, *p* < 0.001]; the genotype by treatment interaction was also significant [F(5,96) = 7.953, *p* < 0.001]. (**c**) LSD-stimulated retrograde walking in βArr2 subjects. A two-way ANOVA found significant treatment effects [F(5,96) = 13.028, *p* < 0.001]; the genotype by treatment interaction was also significant [F(5,96) = 5.199, *p* < 0.001]. (**d**) LSD-stimulated nose poking in WT and βArr2-KO mice. A two-way ANOVA observed significant treatment effects [F(5,125) = 7.512, *p* < 0.001]; the genotype by treatment interaction was also significant [F(5,125) = 4.769, *p* = 0.001]. N = 8–12 mice/group for head twitch, grooming, and retrograde walking; N = 10–15 mice/group for nose-poking. *p < 0.05, ***p ≤ 0.001, WT vs. KO; ^+^*p* < 0.05, ^++^*p* < 0.01, ^+++^*p* ≤ 0.001, LSD vs. indicated groups within genotype; ^^^^*p* < 0.01, ^^^^^*p* < 0.001, 0.05MDL + LSD vs. indicated groups within genotype; ^††^*p* < 0.01, ^†††^*p* < 0.001, 0.1MDL + LSD vs. indicated groups within genotype.

In contradistinction to βArr1 mice, genotype differences were present for βArr2 animals. HTRs were significantly increased in the LSD and 0.05 mg/kg MDL plus LSD groups of WT relative to βArr2-KO mice (*p *values < 0.001) (Fig. 4a). In WT mice, HTRs were stimulated by LSD and they were still enhanced when 0.05 or 0.1 mg/kg MDL were given with LSD relative to the vehicle and MDL controls (*p *values < 0.001). Notably, both 0.1 and 0.5 mg/kg MDL significantly reduced the LSD-stimulated responses (*p *values ≤ 0.002)—with the higher MDL dose being the more efficacious in suppressing HTRs to control levels (*p* < 0.001). In βArr2-KO mice, the LSD and the 0.05 and 0.1 mg/kg MDL plus LSD treatments increased HTRs compared to the vehicle and MDL controls (*p *values ≤ 0.023). Only 0.5 mg/kg MDL was sufficient to normalize the LSD-stimulated response to control levels in the βArr2-KO mice (*p* = 0.019).

For grooming, the durations of responding were higher in WT than in the βArr2-KO groups administered LSD alone, 0.05 mg/kg MDL plus LSD, or 0.5 mg/kg MDL with LSD (*p *values ≤ 0.016) (Fig. 4b). In WT mice, grooming was augmented in the LSD and the 0.05 mg/kg MDL plus LSD groups relative to the vehicle and MDL controls (*p *values < 0.001). While 0.05 mg/kg MDL failed to block the LSD effects, both of the 0.1 and 0.5 mg/kg doses were efficacious in normalizing the responses to that of the controls (*p *values < 0.001). In βArr2-KO animals, the duration of grooming to LSD was not significantly different from the vehicle and MDL controls. Nevertheless, grooming was enhanced in mice administered 0.05 mg/kg MDL plus LSD compared to all groups (*p *values ≤ 0.013), except those given LSD alone.

Since LSD can induce alterations in tactile perception^42^, we examined grooming in detail as it has a chained organization of responses in rodents^43^. Note, since in our video recordings the WT mice in the βArr1 and βArr2 strains responded similarly to the vehicle and MDL controls, as well as to LSD and the 0.5 mg/kg MDL plus LSD treatments for grooming, recordings from only one of the WT strains is presented. Analyses of the video-recordings confirmed that all genotypes engaged in a normal sequence of grooming beginning with the face, progressing down the body, and ending at the feet or tail (Movie 1). When LSD was administered, the sequence of grooming in the WT and βArr1-KO mice became abbreviated, non-sequential, and/or restricted to one area of the body (Movies 2–3). By comparison, the grooming sequence was complete and rarely perturbed with LSD in the βArr2-KO animals (Movie 4). When the 5-HT2AR antagonist MDL was administered alone, the organization of grooming was intact in the WT and βArr1-KO mice (Movie 5). By comparison, with MDL the βArr2-KO animals often paused in grooming bouts and/or displayed twitching of the neck and back muscles; however, they typically finished the grooming sequence (Movie 6). The patterns of grooming among the genotypes administered MDL plus LSD were divergent. In WT mice given MDL plus LSD, the organization of grooming was restored but with some focus initially on facial groming (Movie 7). When the βArr1 mutants received the same treatment, they began the grooming sequence, engaged in focal grooming of a part of the body, and then completed the sequence (Movie 8). When this same drug combination was administered to βArr2-KO mice, they usually began the sequence appropriately, but at some mid- or later-point they would become focused on one body area of grooming and sometimes did not complete the grooming sequence (Movie 9).

Aside from abnormalities in the organization of grooming, LSD also induced retrograde walking and stimulated nose-poking behaviors. Incidences of retrograde walking were increased significantly in WT mice in the groups given LSD or 0.05 MDL plus LSD compared to the βArr2-KO groups (*p *values ≤ 0.040) (Fig. 4c). In WT mice, LSD potentiated the incidences of retrograde walking compared to the MDL and vehicle controls (*p* < 0.001). Although 0.05 mg/kg MDL was ineffective in decreasing this LSD-stimulated behavior, both 0.1 and 0.5 mg/kg MDL suppressed this response to that of controls (*p *values < 0.001). By contrast, LSD was without any significant effect on retrograde walking in the βArr2-KO animals compared to its vehicle and MDL controls.

Similar to retrograde walking, nose-poking behavior was increased by LSD in WT relative to βArr2-KO mice (*p* < 0.001) (Fig. 4d). In WT mice, LSD stimulated nose-poking behaviors relative to all other groups (*p *values < 0.007). All doses of the 5-HT2AR antagonist reduced the LSD-stimulated nose poking to the levels of the vehicle and MDL controls. No treatment effects were noted among the βArr2-KO animals.

In summary, responses to LSD across these LSD-stimulated behaviors were similar between the WT and βArr1-KO mice and the 5-HT2AR antagonist reduced these responses to levels of the vehicle and MDL controls. By contrast, the WT mice responded quite differently from the βArr2-KO animals. HTRs, grooming, retrograde waking, and nose-poking to LSD were significantly higher in WT than in βArr2-KO mice. Notably, LSD disrupted the sequences of grooming in the WT and βArr1-KO mice; βArr2-KO animals were unaffected. Nonetheless, divergent responses to MDL alone or to MDL plus LSD were observed among the genotypes.

### LSD and MDL100907 effects on prepulse inhibition

**Figure 5:**
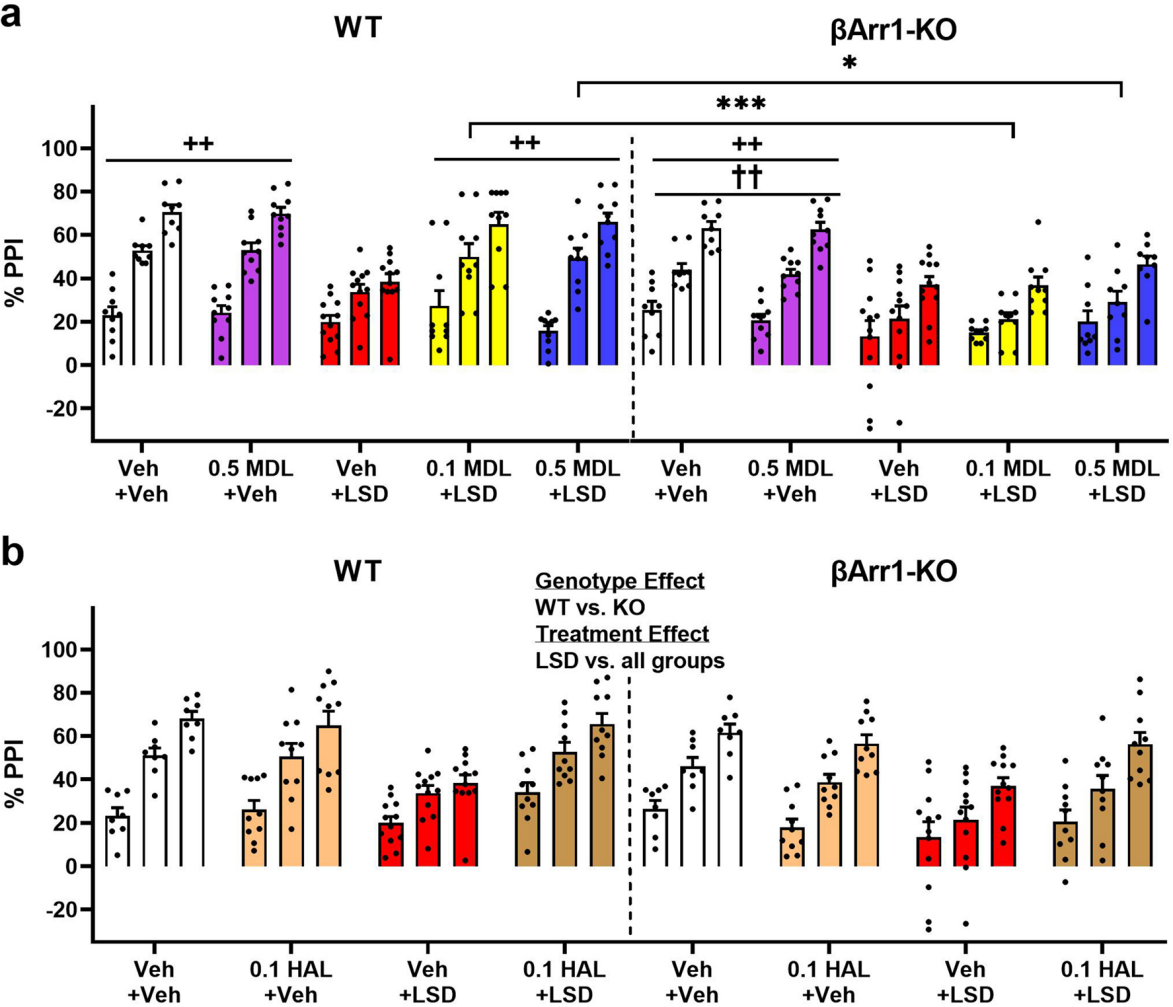
Effects of LSD, MDL100907, and haloperidol on prepulse inhibition in β-arrestin 1 mice. Mice were injected with MDL100907, haloperidol, or the vehicle and administered subsequently the vehicle or LSD prior to testing PPI. (**a**) PPI in WT and βArr1-KO mice treated with MDL or LSD. A RMANOVA found the main effects of prepulse intensity [F(1,91) = 487.507, *p* < 0.001], genotype [F(1,91) = 25.358, *p* < 0.001], and treatment [F(4,91) = 11.435, *p* < 0.001] to be significant. The prepulse intensity by genotype [F(1,91) = 9.162, *p* = 0.003], prepulse intensity by treatment [F(4,91) = 7.944, *p* < 0.001], genotype by treatment [F(4,91) = 2.394, *p* = 0.052], and prepulse intensity by genotype by treatment interactions [F(4,91) = 2.611, *p* = 0.041] were significant. (**b**) PPI in WT and βArr1-KO mice that received haloperidol or LSD. A RMANOVA detected significant main effects for prepulse intensity [F(1,72) = 415.876, *p* < 0.001], genotype [F(1,72) = 7.563, *p* = 0.008], and treatment [F(3,72) = 9.591, *p* < 0.001]. The prepulse intensity by treatment interaction was also significant [F(3,72) = 7.702, *p* < 0.001]. N = 8–12 mice/group. *p < 0.05, ***p ≤ 0.001, WT vs. KO; ^++^*p* < 0.01, LSD vs. indicated groups within genotype; ^††^*p* < 0.01, 0.1MDL + LSD vs. indicated groups within genotype.

LSD disrupts PPI in both rats and humans and the response can be restored with 5-HT2AR antagonists^37,44^. βArr1 mice were pre-treated with the vehicle or with 0.1 or 0.5 mg/kg MDL. Subsequently, they were administered the vehicle or 0.3 mg/kg LSD and tested in PPI. No significant genotype or treatment effects were observed for null activity or in response to the 120 dB startle stimulus (Supplementary Figure S3a-b). In contrast, genotype effects were found in PPI where responses in the WT groups that received 0.1 or 0.5 mg/kg MDL plus LSD were higher than those in βArr1-KO animals (*p *values ≤ 0.018) (Fig. 5a). As anticipated, LSD disrupted PPI in both βArr1 genotypes relative to their vehicle and 0.5 mg/kg MDL controls (*p *values ≤ 0.002). Both 0.1 and 0.5 mg/kg MDL normalized PPI in WT mice to control levels. In βArr1-KO animals, PPI was still significantly disrupted in mice administered 0.1 mg/kg MDL with LSD relative to controls (*p *values ≤ 0.001). Although PPI responses in the 0.5 mg/kg MDL plus LSD group were not significantly different from these controls, they were also not significantly different from the LSD group. Hence, LSD disrupted PPI in both WT and βArr1-KO mice, while MDL restored PPI only in WT animals.

Since haloperidol can normalize PPI in mouse models^45^, we tested whether this antipsychotic drug could normalize the LSD-disrupted PPI in the βArr1-KO mice. Overall treatment effects were found where null activities were higher in the 0.1 mg/kg haloperidol plus LSD group than in mice treated with the vehicle or haloperidol alone (*p *values = 0.009) (Supplementary Figure S3c). An assessment of startle activity revealed that responses were lower overall in the WT relative to βArr1-KO mice (*p* = 0.028) (Supplementary Figure S3d). For PPI, genotype effects were found where responses were reduced overall in the βArr1-KO compared to the WT animals (*p* = 0.008) (Fig. 5b). Treatment effects were observed also, where LSD suppressed PPI relative to all other treatment conditions (*p *values ≤ 0.002). Here, haloperidol normalized the LSD-disrupted PPI to control levels in both WT and βArr1-KO mice.

**Figure 6:**
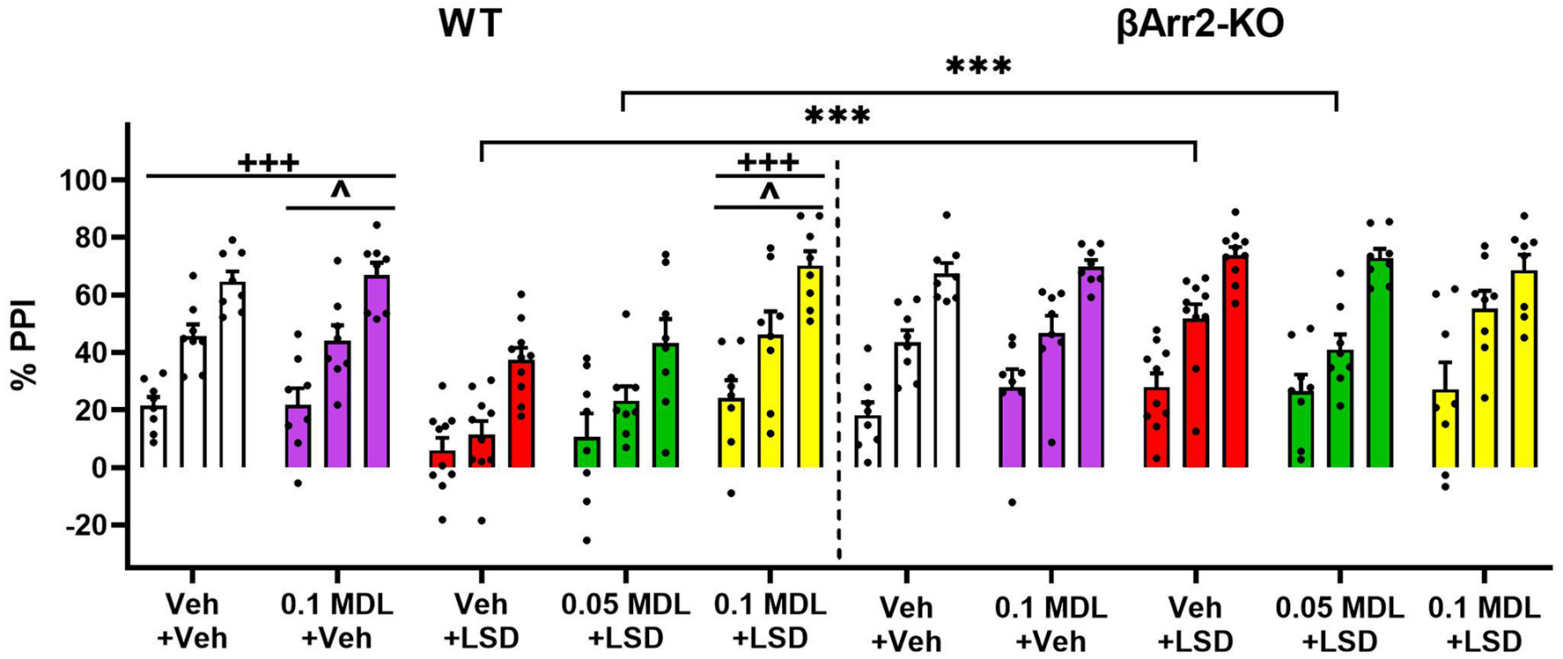
Effects of LSD and MDL100907 on prepulse inhibition in β-arrestin 2 mice. A description of the experimental design is provided in the Fig. 5 legend. PPI in WT and βArr2-KO mice treated with MDL or LSD. A RMANOVA found the main effects of prepulse intensity [F(1,74) = 580.044, *p* < 0.001], genotype [F(1,74) = 18.823, *p* < 0.001], and treatment [F(4,74) = 3.953, *p* = 0.006] to be significant; the genotype by treatment interaction [F(4,74) = 5.660, *p* < 0.001] was also significant. N = 8–10 mice/group. ***p < 0.05, WT vs. KO; ^+++^*p* < 0.001, LSD vs. indicated groups within genotype; ^^^*p* < 0.05, 0.05MDL + LSD versus indicated groups within genotype.

PPI responses in the βArr2 mice were examined also. Overall null activity was decreased in the 0.1 mg/kg MDL plus LSD group compared to the vehicle control and the LSD group (*p *values ≤ 0.003) (Supplementary Figure S4a). No significant effects were detected for startle activity (Supplementary Figure S4b). Nevertheless, genotype differences were evident for PPI (Fig. 6). Here, responses to LSD and to the 0.05 MDL plus LSD treatments were reduced in WT relative to the βArr2-KO mice (*p *values ≤ 0.001). In WT animals, LSD disrupted PPI compared to the MDL and vehicle controls (*p *values = 0.001). PPI remained disrupted in the 0.05 mg/kg MDL plus LSD group relative to the MDL control (*p* = 0.050). However, PPI was normalized to controls with 0.1 mg/kg MDL. By comparison, LSD was without effect in the βArr2-KO mice. Collectively, these findings show that LSD disrupts PPI in both genotypes of the βArr1 mice. PPI was disrupted also with LSD in the WT animals from the βArr2 strain. The 5-HT2AR antagonist restored PPI in both WT strains, whereby haloperidol was required to normalize it in βArr1-KO mice. By contrast, PPI in βArr2-KO mice was unaffected by LSD.

### Effects of *Arrb1* or *Arrb2* deletion on 5-HT2AR expression

**Figure 7:**
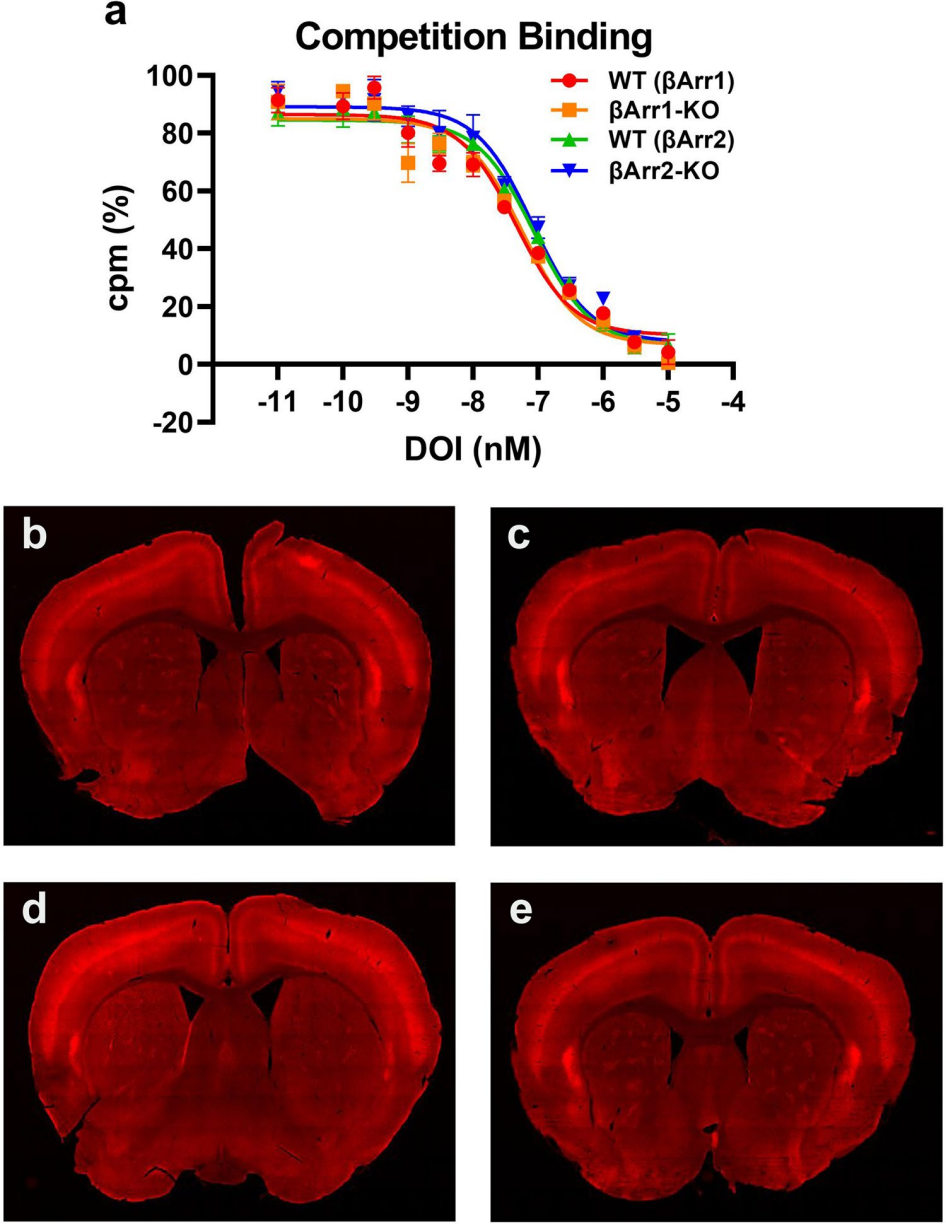
Radioligand binding and immunohistochemistry of 5-HT2ARs in βArr1 and βArr2 mice. (**a**) Competition binding with [^3^H]-ketanserin using membranes from βArr1 and βArr2 brains. The K_d_ values for binding were 21.3, 26.8, 40.8, and 38.7 nM from WT and βArr1-KO, and WT and βArr2-KO mice, respectively. N = 3 mice/genotype. (**b-e**) Representative 5-HT2AR immunofluorescence in coronal brain sections from respective WT and βArr1-KO mice (*top*), and WT and βArr2-KO (*bottom*) animals.

We examined whether deletion of *Arrb1* or *Arrb2* could alter 5-HT2AR expression by radioligand binding with brains from WT and βArr1-KO, and WT and βArr2-KO littermates. When [^3^H]-ketanserin competition binding was examined, displacement with DOI and Ki values were found to be very similar with membranes from the WT and βArr1-KO and the WT and βArr2-KO brains (Fig. 7a). We examined also 5-HT2AR immunofluorescence in βArr1 and βArr2 brain sections (Fig. 7b–e). Here, we detected no apparent alterations in the relative receptor distributions among the genotypes, with prominent 5-HT2AR immunostaining in the cortex. Together, these results are consistent with the hypothesis that neither global *Arrb1* nor global *Arrb2* genetic deletion *decreases* 5-HT2AR expression.

## Discussion

In the present study, we analyzed whether global deletion of *Arrb1* or *Arrb2* was involved in LSD-stimulated responses in mice. In many cases, we found that LSD modified behaviors in both βArr strains of WT mice, as well as in the βArr1-KO animals. By contrast, LSD exerted little effect on βArr2-KO responses. Collectively, these results suggest the LSD-stimulated responses require βArr2. In this regard, βArr2 is reported to play a similar role in morphine-stimulated hyperlocomotion^46^ and amphetamine-stimulated locomotor and rearing activities in βArr2 mice^47^.

While we found LSD stimulates locomotion in mice, in rats it has been reported to decrease ambulation^35^ or increase locomotion^32,33,36^. While an inhibitory response to 0.2 mg/kg LSD was observed in rats, we only saw stimulatory effects with 0.3 mg/kg LSD and in pilot studies, doses of 0.1–0.5 mg/kg LSD were all stimulatory. An absence of LSD inhibitory effects could be attributed to differences in species tested, test environment and apparatus, and/or test procedure. In humans LSD’s behavioral effects can be context specific^1,2^ and our 30 min habituation to the open field prior to LSD administration may have reduced emotionality in our mice, such that only the stimulatory effects of LSD were evident.

To determine whether the locomotor-stimulating effects of LSD were due to 5-HT2AR activation, MDL was used as an antagonist. When used alone, this antagonist exerted no effects on motor performance in either βArr mouse strain. Importantly, 0.1 and 0.5 mg/kg MDL blocked the locomotor-stimulating effects of LSD in both WT strains and in the βArr1-KO animals. A similar antagonist effect has been observed in rats^36^. Hence, the present results indicate that the LSD-induced hyperactivity in βArr mice is promoted through the 5-HT2AR. Despite this finding, LSD binds to other GPCRs including dopamine receptors^8^. Since various drugs of abuse are known to stimulate dopamine neurotransmission, it is likely these receptors are involved in the observed LSD-stimulated response. Notably, global deletion of *Arrb2* blunts locomotor responses to amphetamine in the open field^47,48^. Thus, the reduced response to LSD by the βArr2-KO mice may be due actions mediated not only through the 5-HT2AR, but also through a dopamine receptor mechanism.

Besides motor activity, we examined the effects of LSD on HTRs, grooming, retrograde walking, and nose-poking behaviors. LSD and other psychedelics are well-known to stimulate HTRs in mice^17,38,41^ and this behavior has been proposed as a proxy for hallucinations in humans^12^. Compared to vehicle, LSD stimulated HTRs to similar extents in WT and βArr1-KO mice. In βArr2-KO animals, this response was severely blunted compared to the WT controls. These results were unexpected since the individual competition binding curves could be superimposed among the different genotypes. Regardless, in both βArr1 and βArr2 mice, MDL reduced HTRs to levels of the vehicle controls. These findings are consistent not only with the known action of MDL on blocking HTRs to various hallucinogens^14–16^, but also on the inability of LSD and other psychedelics to induce this response in the *htr2A* homozygous mutant mice^17,18,38^.

Aside from HTRs in rodents, LSD accentuates grooming behaviors in cats^49^ and it can stimulate or inhibit grooming in mice^39,40^. In our investigations, LSD augmented grooming in both WT strains, and in βArr1-KO animals. By comparison, this psychedelic was ineffective in βArr2-KO mice. In both WT strains and in βArr1-KO animals, 0.1 and 0.5 mg/kg MDL returned the LSD-stimulated grooming to control levels. Thus, antagonism of the 5-HTR2A was sufficient to restore LSD-induced grooming to baseline.

Effects of LSD were examined also for the organization of grooming behavior. Under vehicle treatment, all mice displayed similar patterns of grooming that began with the face, progressed to the flanks, and ended with the feet or tail. LSD disturbed this sequence of events in both WT strains and in βArr1-KO mice. By comparison, grooming in the βArr2-KO mice was largely unaffected by LSD. MDL did not alter grooming in the WT and βArr1-KO mice, whereas it prolonged grooming and promoted twitching of the neck and back muscles in βArr2-KO animals. This 5-HT2AR antagonist blocked the LSD-disrupting effects on the organization of grooming in WT mice and it mostly restored it in βArr1-KO animals. The MDL-LSD combination in βArr2-KO animals produced some disturbances, but the mice typically completed the grooming sequence. Together, these results suggest that additional receptor systems may be involved in the LSD-induced grooming responses.

The effects of LSD on retrograde walking and nose-poking responses were also examined. We found LSD to stimulate these behaviors in WT animals from both strains, as well as in the βArr1-KO mice. However, LSD promoted neither response in βArr2-KO animals. Nevertheless, in the other genotypes MDL restored retrograde walking and nose-poking to the levels of vehicle controls. Hence, this 5-HT2AR antagonist normalized these LSD-stimulated behaviors. It should be emphasized that repetitive behaviors like grooming or nose-poking may be mediated by several receptor systems. For instance, these repetitive responses can be modified at least through alterations in serotonergic, dopaminergic, and glutamatergic neurotransmission^50,51^. Given the polypharmacology of LSD^8^, it is likely the LSD disruptive effects on these behaviors are mediated through additional receptor systems in our experiments.

LSD-induced states share many similarities with the early acute phases of psychosis^2^. PPI is abnormal in individuals diagnosed with schizophrenia^52^ and LSD disrupts PPI in rats^36,39,44^. In βArr1 mice, LSD disrupted PPI in both genotypes without affecting startle or null activities. Both 0.1 and 0.5 mg/kg MDL restored the LSD-disrupted PPI, but only in WT mice; an effect consistent with the action of the 5-HT2AR antagonist MDL11939 in rats^44^. By comparison, MDL was ineffective in blocking the LSD effects in βArr1-KO animals. Since LSD activates human dopamine D2 receptors^8,53^, we used haloperidol as a D2 antagonist. We found this antagonist to restore the LSD-disrupted PPI in the βArr1-KO mice. Parenthetically, both 0.1 and 0.2 mg/kg haloperidol failed to rescue PPI in rats given 0.1 mg/kg LSD (s.c.)^36^; the possible reasons for this discrepancy in mice versus rats are unclear. When βArr2 mice were tested, LSD disrupted PPI selectively only in WT mice. Notably, βArr2-KO mice were completely unresponsive to this psychedelic. As with WT animals from the βArr1 strain, MDL also normalized the LSD-disrupted PPI in the WT βArr2 mice. Thus, the LSD effects on PPI in the βArr mice are complex, with restoration of PPI with MDL in both strains of WT mice, normalization of PPI with haloperidol in βArr1-KO animals, and without any discernable effect in βArr2-KO subjects.

LSD and other psychedelics are well-known for their hallucinogenic actions^1^ and these responses have been attributed to 5-HT2AR agonism^11,17^. We observed LSD to stimulate motor activity, head twitches, grooming, retrograde walking, and nose-poking in both βArr strains of WT mice and in βArr1-KO animals. LSD also disrupted PPI in these same genotypes. The LSD-elicited responses in βArr2-KO mice were either significantly attenuated or completely absent. In conditions where LSD produced changes in behavior, these alterations were blocked with the 5-HT2AR antagonist MDL. While these results suggest that the 5-HT2AR is an essential component for all these responses, it should be recalled that LSD exerts a plethora of actions at many GPCRs^8–10^ and, aside from HTRs, other behaviors are inconsistently affected by hallucinogens^17^. Hence, it is likely that LSD’s effects on the 5-HT2AR are involved in a cascade of many GPCR-signaling events mediating these varied responses. In this regard, arrestins are known to serve as scaffolds for many signal transduction molecules^47,54^. Future work will examine some of these mechanisms across our behavioral tests.

Our immunohistochemical results show that the 5-HT2AR protein is expressed in several areas of the brain, especially the cortex. In this brain region the 5-HT2ARs are localized primarily to pyramidal cells and, to some extent, to interneurons^55^. Since the glutamatergic neurons project to multiple subcortical brain areas, actions on 5-HT2ARs in these neurons could exert varied effects on behavior. Within 5-HT2AR-containing neurons, agonist actions at this receptor can lead to G protein-dependent and -independent signaling, the latter of which involves βArr^22–24^. Disruption of the *Arrb1* or *Arrb2* genes would leave G protein signaling intact, while affecting respective βArr2 or βArr1 mediated signaling and desensitization. Zurkovsky and colleagues^48^ have proposed a model of arrestin actions that may apply to our results with LSD. Both βArr1 and βArr2 are co-expressed, with few exceptions throughout the adult rodent brain. However, expression of βArr1 mRNA is much higher than that for βArr2–-except in selected brain areas^56^. While the 5-HT2AR binds to both βArr proteins in vitro and is complexed with these βArrs in cortical neurons *in vivo*^24^, there is some evidence that the affinities of βArr1 and βArr2 for different GPCRs can vary *in vitro*^47,57^. Moreover, in the few systems that have been studied, signaling in the presence of βArr2 is more efficacious than with βArr1^47^. In our experiments, the LSD-elicited responses were largely intact in the βArr1-KO than in the βArr2-KO mice, because in the βArr1-KO animals βArr2-mediated signaling is still retained. In this regard, it is especially intriguing that LSD-induced HTRs were much more robust in both WT strains and in the βArr1-KO animals, than in the βArr2-KO mice. Our results with LSD suggest that βArr2 may be essential for the expression of hallucinogenic-like actions at the 5-HT2AR.

## Methods

### Subjects

Adult male and female WT and βArr1-KO, and WT and βArr2-KO mice were used in these experiments^30,31^. All mice had been backcrossed onto a C57BL/6J genetic background. Heterozygotes were used to generate the respective WT and KO animals. The mice were housed 3–5/cage in a temperature- and humidity-controlled room on a 14:10 h (lights on at 0600 h) light–dark cycle with food and water provided ad libitum. All experiments were conducted with an approved protocol from the Duke University Institutional Animal Care and Use Committee and all experiments and methods were performed in accordance with the relevant regulations and ARRIVE guidelines.

### Drugs

The drugs consisted of (+)-LSD-(+)-tartrate (NIDA Drug Supply Program, Bethesda, MD), MDL 100,907 (Bio-Techne Corp., Minneapolis, MN), haloperidol (Sigma-Aldrich, St. Louis, MO), and (-)-1-(2,5-diethoxy-4-iodophenyl)-2-aminopropane hydrochloride (DOI; Sigma-Aldrich). The vehicle was composed of N,N-dimethyllacetamide (final volume 0.5%; Sigma-Aldrich) that was brought to volume with 5% 2-hydroxypropoyl-β-cyclodextrin (Sigma-Aldrich) in water (Mediatech Inc., Manassas, VA). All drugs were administered (i.p.) in a 5 mL/kg volume. All studies used groups that were administered the vehicle and the 5-HT2AR antagonist, MDL100907 as controls.

### Open field activity

Motor activities were assessed in an open field (21 × 21 × 30 cm; Omnitech Electronics, Columbus, OH) illuminated at 180 lux^45^. All behaviors were filmed. Mice were injected with the vehicle or different doses of MDL and placed into the open field. Thirty min later, they were administered the vehicle or LSD and were immediately returned to the open field for 90 min. Motor activity was monitored using Fusion Versamax 5.3 Edition software (Omnitech Electronics, Columbus, OH) for locomotor activity (distance traveled), rearing (vertical beam-breaks), and stereotypical activities (repetitive beam-breaks less than 1 s) in 5-min blocks or as cumulative activities.

### Head twitch, grooming, and retrograde walking

These behaviors were filmed during assessment of motor activity. The responses were scored over the first 30 min following injection of the vehicle or LSD after collection of baseline activity. Observers who were blinded to the sex, genotype, and treatment conditions in the experiments scored the video recordings. The data are expressed as the numbers of head twitches, duration of grooming, and incidences of retrograde walking.

### Nose-poking responses

Nose-pokes were monitored in a 5-choice serial reaction-time apparatus (Med Associates Inc., St. Albans, VT)^58^. Each chamber had five LED-illuminated 1.24 cm^2^ nose-poke apertures with infrared diodes to register nose pokes. No food or liquid reward was available. Mice were injected with the vehicle or different doses of MDL and returned to their home-cages. Thirty min later, the animals were injected with the vehicle or LSD and were placed immediately into the operant chambers for 30 min. The data are depicted as the numbers of head pokes.

### Prepulse inhibition (PPI)

PPI of the acoustic startle response was conducted using SR-LAB chambers (San Diego Instruments, San Diego, CA) as reported^45^. Mice were injected with vehicle or different doses of MDL or with 0.1 mg/kg haloperidol and returned to their home cages. Fifteen min later the animals received the vehicle or LSD and were placed into the apparatus. After 10 min of habituation to a white noise background (64 dB), testing began. Each test consisted of 42 trials with 6 null trials, 18 pulse-alone trials, and 18 prepulse-pulse trials. Null trials comprised the white noise background, pulse trials consisted of 40 ms bursts of 120 dB white-noise, and prepulse-pulse trials were composed of 20 ms pre-pulse stimuli that were 4, 8, or 12 dB above the white-noise background (6 trials/dB), followed by the 120 dB pulse stimulus 100 ms later. Testing commenced with 10 pulse-alone trials followed by combinations of the prepulse-pulse and null trials, and it terminated with 10 pulse-alone trials. PPI responses were calculated as %PPI = [1 − (pre-pulse trials/startle-only trials)]*100.

### Radioligand binding and immunohistochemistry of the 5-HT2AR

Binding experiments on mouse brains were conducted as described using 2.3 nM [^3^H]-ketanserin (NEN Life Sciences, Wellesley, MA) as the radioligand^53^ with varying concentrations of unlabeled DOI (Sigma-Aldrich) and 75 μg protein from brain. Binding was analyzed by GraphPad Prism (San Diego, CA). The 5-HT2AR immunofluorescence study was performed as described^59^ with a validated 5-HT2AR-specific antibody^60^. Mice were intracardially perfused with PBS followed by 4% paraformaldehyde (PFA) in PBS. Brains were harvested, post-fixed overnight in 4% PFA, and dehydrated in 30% sucrose. Brains were sectioned at 40 µm by cryostat. Brain sections were washed 3X with 0.4% Triton X-100 in PBS (TX-100/PBS) before incubating for 1 h with blocking buffer (5% normal donkey serum in 0.4% TX-100/PBS). Next, they were incubated for 48 h at 4 °C with the anti-5-HT2AR antibody (1:250, #RA24288; Neuromics, Edian, MN). Subsequently sections were washed 3X with 0.1% TX-100/PBS and incubated for 2 h with the secondary antibody (1:1000, donkey anti-rabbit, Alexa Fluor 594; Jackson Immunoresearch, West Grove, PA). The sections were imaged under a 20X objective using an Olympus VS120 virtual slide microscope (Olympus, Tokyo, Japan).

### Statistics

All statistical analyses were performed with IBM SPSS Statistics 27 programs (IBM, Chicago, IL). The data are presented as means and standard errors of the mean. No sex effects were detected in any experiments. Hence, this variable was collapsed. All data were normally distributed. One- or two-way ANOVA, repeated measures ANOVA (RMANOVA), or analyses of covariance (ANCOVA) were used to analyze the data, followed by Tukey or Bonferroni *post-hoc* analyses. A *p* < 0.05 was considered significant. All results were plotted using GraphPad Prism.

## Supplementary Information

Supplementary Information 1.

Supplementary Movie 1.

Supplementary Movie 2.

Supplementary Movie 3.

Supplementary Movie 4.

Supplementary Movie 5.

Supplementary Movie 6.

Supplementary Movie 7.

Supplementary Movie 8.

Supplementary Movie 9.

## Data Availability

Data that support this study are available from the corresponding authors upon reasonable request.
