# Supplementary material for: LSD-stimulated behaviors in mice require β-arrestin 2 but not β-arrestin 1
Source: Sci Rep. 2021 Sep 3;11:17690. doi: 10.1038/s41598-021-96736-3 (PMC8417039; doi:10.1038/s41598-021-96736-3)
Supplement: Supplementary file 1 — Supplementary Information 1. [file 41598_2021_96736_MOESM1_ESM.pdf]

Supplementary Information

Supplementary tables

Supplementary Table S1. Cumulative baseline locomotor, rearing, and stereotypical activities in β-arrestin 1 mice.

**Motor Response**	**WT[a]**	**βArr1-KO[a]**
Distance Travelled		
Vehicle	1033.6 ± 88.35	978.9 ± 93.79
0.5 mg/kg MDL - Vehicle	853.1 ± 89.82	820.7 ± 29.29
Vehicle + 0.3 mg/kg LSD	984.4 ± 63.07	926.8 ± 59.14
0.1 mg/kg MDL + 0.3 mg/kg LSD	831.2 ± 49.49	783.1 ± 96.35
0.5 mg/kg MDL + 0.3 mg/kg LSD	819.1 ± 42.20	878.9 ± 107.56
Vertical Activity		
Vehicle	393.4 ± 60.03[b]	267.4 ± 69.58[b]
0.5 mg/kg MDL - Vehicle	211.3 ± 33.01	364.4 ± 72.51
Vehicle + 0.3 mg/kg LSD	363.9 ± 79.27[b]	317.6 ± 41.56[b]
0.1 mg/kg MDL + 0.3 mg/kg LSD	199.0 ± 20.55[b]	113.4 ± 25.17[b]
0.5 mg/kg MDL + 0.3 mg/kg LSD	193.0 ± 28.21[b]	117.7 ± 22.31[b]
Stereotypical Activity		
Vehicle	4244.6 ± 379.95[c]	4004.3 ± 460.29[c]
0.5 mg/kg MDL - Vehicle	3227.9 ± 502.66	3542.4 ± 221.92
Vehicle + 0.3 mg/kg LSD	4047.6 ± 328.81[c]	3556.0 ± 311.30[c]
0.1 mg/kg MDL + 0.3 mg/kg LSD	2643.0 ± 246.27[c]	2335.9 ± 234.63[c]
0.5 mg/kg MDL + 0.3 mg/kg LSD	3366.8 ± 164.17[c]	2571.4 ± 281.79[c]

[a]N = 8-17 mice/genotype/treatment.
[b]For vertical activity, a two-way ANOVA found pre-assignment to treatment conditions to be significant [$F(4,93)=6.943$, $p<0.001$]. To control for this effect, ANCOVA was used to analyze the post-injection results.
[c]For stereotypical activity, a two-way ANOVA found pre-assignment to treatment conditions to be significant [$F(4,93)=7.110$, $p<0.001$]. To control for this effect, ANCOVA was used to analyze the post-injection results.

Supplementary Table S2. Cumulative baseline locomotor, rearing, and stereotypical activities in β-arrestin 2 mice.

**Motor Response**	**WT**[a]	**βArr2-KO**[a]
Distance Travelled		
Vehicle	489.34 ± 80.06	346.74 ± 49.35
0.5 mg/kg MDL - Vehicle	256.78 ± 78.79	315.93 ± 57.03
Vehicle + 0.3 mg/kg LSD	519.58 ± 125.65	314.36 ± 44.71
0.05 mg/kg MDL + 0.3 mg/kg LSD	407.60 ± 60.61	310.51 ± 45.67
0.15 mg/kg MDL + 0.3 mg/kg LSD	470.92 ± 109.35	454.93 ± 58.01
0.5 mg/kg MDL + 0.3 mg/kg LSD	296.28 ± 46.81	381.95 ± 74.91
Vertical Activity		
Vehicle	50.80 ± 18.22	24.00 ± 6.35
0.5 mg/kg MDL - Vehicle	11.63 ± 4.28	12.62 ± 5.07
Vehicle + 0.3 mg/kg LSD	39.41 ± 10.58	22.30 ± 5.39
0.05 mg/kg MDL + 0.3 mg/kg LSD	36.12 ± 12.09	31.62 ± 9.53
0.1 mg/kg MDL + 0.3 mg/kg LSD	36.00 ± 12.51	63.37 ± 24.12
0.5 mg/kg MDL + 0.3 mg/kg LSD	23.12 ± 9.20	38.62 ± 14.32
Stereotypical Activity		
Vehicle	1428.10 ± 178.43	1197.40 ± 88.91
0.5 mg/kg MDL - Vehicle	865.37 ± 188.64	960.13 ± 135.06
Vehicle + 0.3 mg/kg LSD	1229.25 ± 156.81	1074.20 ± 95.65
0.05 mg/kg MDL + 0.3 mg/kg LSD	1174.13 ± 115.34	1084.75 ± 196.94
0.1 mg/kg MDL + 0.3 mg/kg LSD	1131.40 ± 184.69	1224.12 ± 155.35
0.5 mg/kg MDL + 0.3 mg/kg LSD	1064.12 ± 194.88	1192.75 ± 133.49

[a]N = 8-12 mice/genotype/treatment.

Supplementary Video Clips

Movie 1. Vehicle-treated WT mouse. Representative example of a vehicle-treated WT mouse displaying a full grooming sequence. Here, the mouse grooms its face, left flank, right flank, base of tail, and feet. The vehicle-treated WT mice from both βArr strains and the βArr1-KO and βArr2-KO mice show similar responses.

Movie 2. LSD-treated WT mouse. Representative example of a WT mouse given LSD plus the vehicle that displays a prolonged focal grooming bout. The mouse focuses on grooming its tail. Other WT mice may focus on grooming their flanks or feet, rarely the face. If the face is groomed, this behavior is typically aborted soon afterwards. The LSD-treated WT mice from both βArr strains show similar responses.

Movie 3. LSD-treated βArr1-KO mouse. Representative example of a βArr1-KO mouse administered LSD plus the vehicle that shows disorganized grooming. These mutants typically begin a grooming sequence, focusing on the face and abdomen--where they switch back and forth--before proceeding to the flanks or tail and then they rapidly repeat this sequence.

Movie 4. LSD-treated βArr2-KO mouse. Representative example of a βArr2-KO mouse injected with LSD plus the vehicle. The organization of the grooming sequence in these mice is complete and is rarely abbreviated or disrupted.

Movie 5. MDL-treated WT mouse. Representative example of a WT mouse that received MDL plus the vehicle. The organization of the grooming sequence in the WT and βArr1-KO mice is intact. The MDL plus vehicle-treated WT mice from both βArr strains show similar responses.

Movie 6. MDL-treated βArr2-KO mouse. Representative example of a βArr2-KO mouse that received MDL plus the vehicle. While these mutants display an intact grooming sequence when treated with MDL, they also pause in the grooming bout and display twitching/spasticity of the muscles along the back and neck.

Movie 7. WT mouse treated with MDL plus LSD. Representative example of a WT mouse administered MDL followed by LSD. The MDL appears to restore the organization of grooming in these mice. The MDL plus LSD-treated WT mice from both βArr strains show similar responses.

Movie 8. βArr1-KO mouse treated with MDL plus LSD. Representative example of a βArr1-KO mouse given MDL followed by LSD. These mutants begin the sequence of grooming, but engage in focal grooming on the face and abdomen, changing between these two areas before moving to the flanks and completing the sequence. Note, this pattern of behavior is similar to the LSD plus vehicle sequence, except the mice do not repeat the sequence immediately upon completion.

Movie 9. βArr2-KO mouse treated with MDL plus LSD. Representative example of a βArr2-KO mouse injected with MDL followed by LSD. The organization of grooming is relatively normal except there is some focal grooming mid-point in the sequence and this behavior is not completed beyond the side groom. Hence, MDL given with LSD partially disturbs completion of the grooming sequence in the βArr2-KO mice.

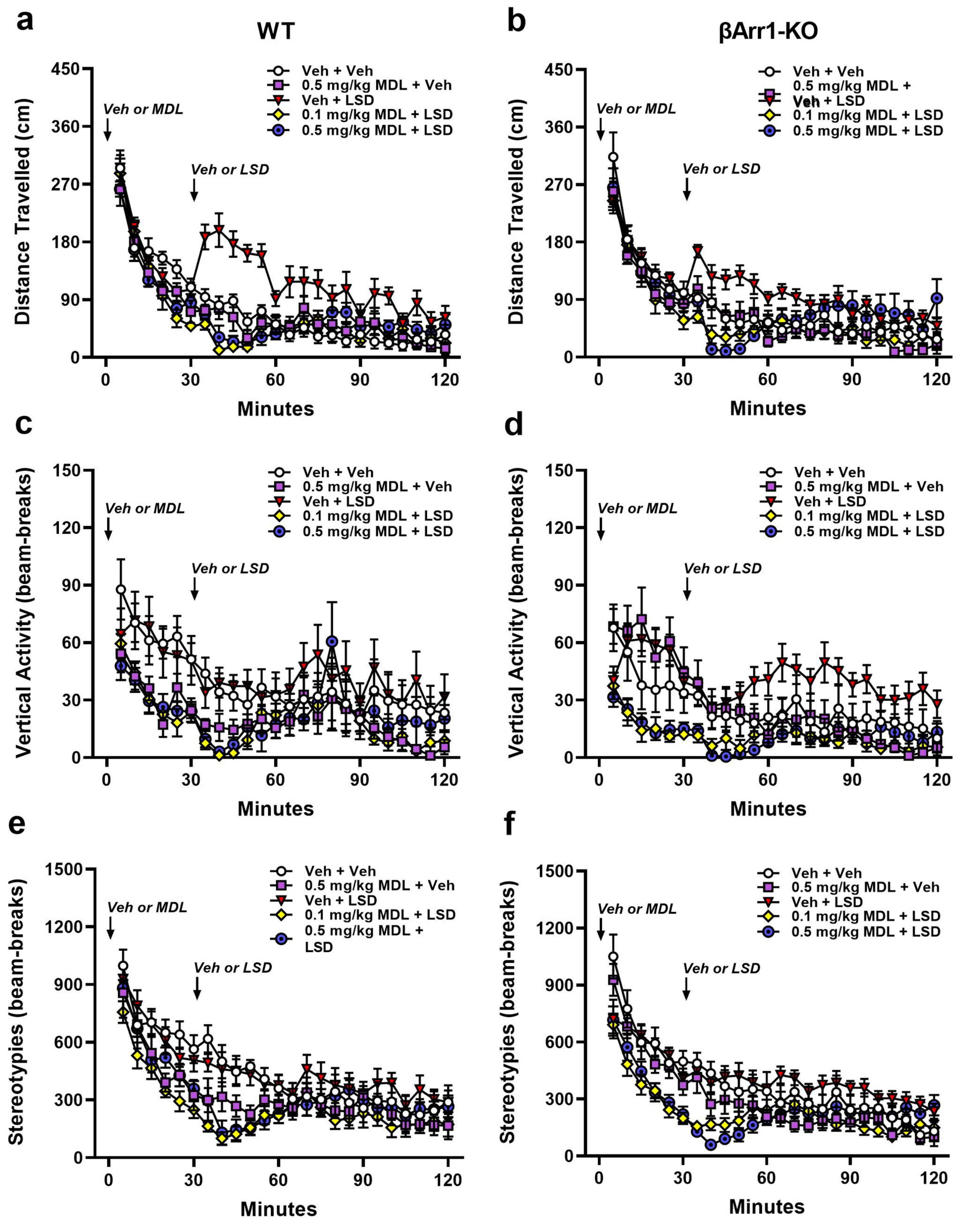

Figure S1. Effects of LSD and MDL100907 on motor activities in 5-min intervals for β-arrestin 1 mice. A description of the experimental design can be found in the legend for Figure 1. The data are presented at 5-min intervals. **a,b** Locomotor activities in WT and βArr1-KO mice. **c,d** Rearing activities in βArr1 animals. **e,f** Stereotypical activities in βArr1 subjects. N = 8-17 mice/group.

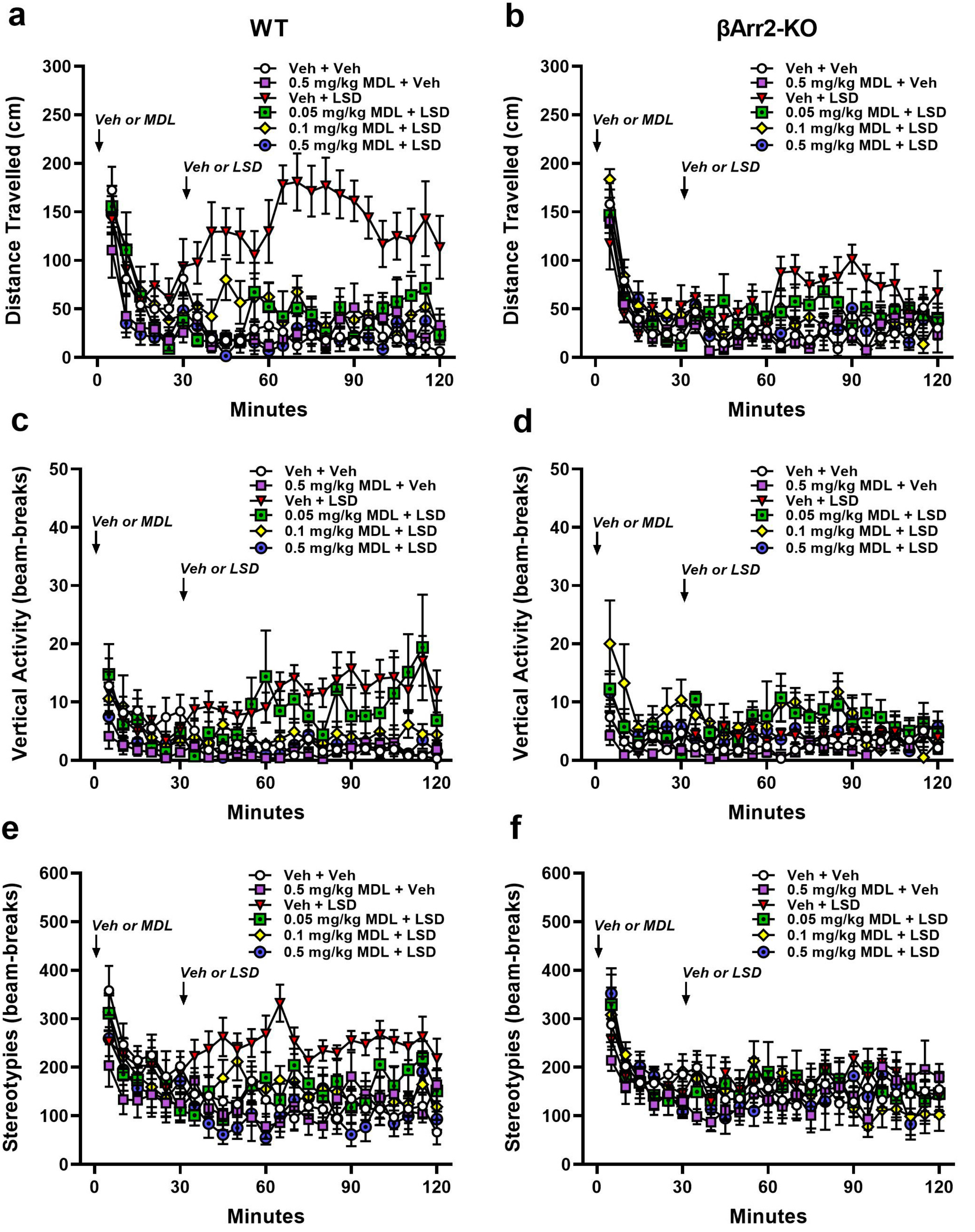

Figure S2. Effects of LSD and MDL100907 on motor activities in 5-min segments for β-arrestin 2 mice. The experimental procedure is described in the Figure 1 legend. Motor activities are displayed at 5-min intervals. **a,b** Locomotor activities in WT and βArr2-KO mice. **c,d** Rearing activities in βArr2 animals. **e,f** Stereotypical activities in βArr2 subjects. N = 8-12 mice/group.

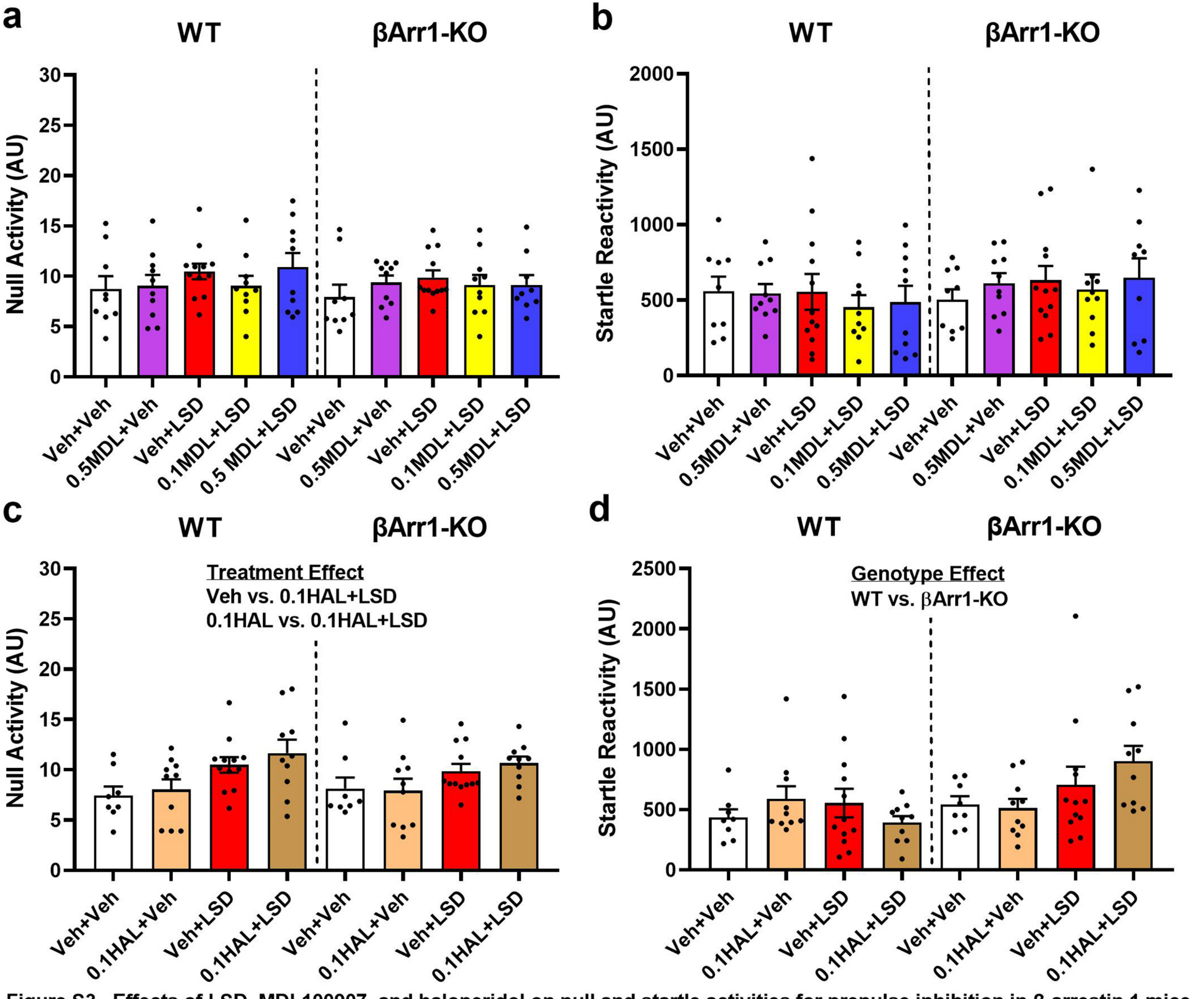

Figure S3. Effects of LSD, MDL100907, and haloperidol on null and startle activities for prepulse inhibition in β-arrestin 1 mice. The experimental procedure is described in the Figure 5 legend. **a,b** Null and startle activities in WT and βArr1-KO mice treated with MDL and LSD. No significant effects were found for null or startle activities. **c,d** Null and startle activities in WT and βArr1-KO mice treated with haloperidol and LSD. A two-way ANOVA for null activity identified a significant treatment effect [$F(3,72)=5.673$, $p=0.002$]. A two-way ANOVA for startle activity detected a significant genotype effect [$F(1,72)=5.015$, $p=0.028$]. N = 8-12 mice/group.

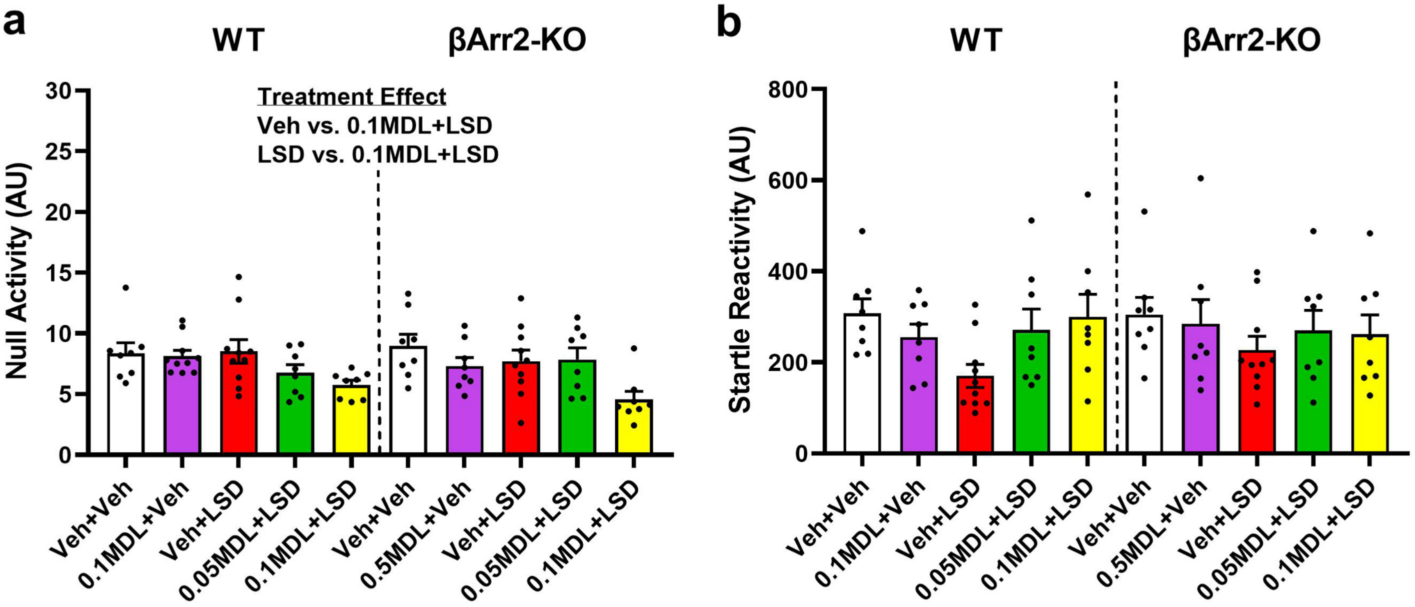

Figure S4. Effects of LSD and MDL100907 on null and startle activities for prepulse inhibition in β-arrestin 2 mice. The experimental procedure is described in the Figure 5 legend. **a,b** Null and startle activities in WT and βArr2-KO mice treated with MDL and LSD. A two-way ANOVA for null activity observed a significant treatment effect [$F(4,74)=5.439$, $p=0.001$]. N = 8-10 mice/group.
